# Some Lessons for the Future from the Global Malaria Eradication Programme (1955–1969)

**DOI:** 10.1371/journal.pmed.1000412

**Published:** 2011-01-25

**Authors:** José A. Nájera, Matiana González-Silva, Pedro L. Alonso

**Affiliations:** 1 Retired (formerly WHO), Crans-Celigny, Vaud, Switzerland; 2 Barcelona Centre for International Health Research (Hospital Clínic, Universitat de Barcelona), Barcelona, Spain; 3 Centro de Investigaçao em Saude da Manhiça, Maputo, Mozambique

## Abstract

Encouraged by the early success of using dichloro-diphenyl-trichloroethane (DDT) against malaria, the World Health Organization (WHO) embarked on the Global Malaria Eradication Program (GMEP) in 1955. Fourteen years later, the campaign was discontinued when it was recognised that eradication was not achievable with the available means in many areas, although the long-term goal remained unchanged. During the GMEP, malaria was permanently eliminated from many regions. In other areas, however, substantial gains were lost in resurgences, sometimes of epidemic proportions. During the 1970s and 1980s, because of economic and financial crises, international support for malaria control declined rapidly, but in the past decade, following increasing demands from endemic countries and promising results from scaling up of control activities, interest in malaria elimination and the long-term goal of eradication has received international political and financial support. In 2007, there was a renewed call for malaria eradication and a consultative process to define a research and development agenda for malaria eradication (malERA) was established. Lessons learned from the GMEP (1955–1969) highlight the fact that no single strategy can be applicable everywhere and that a long-term commitment with a flexible strategy that includes community involvement, integration with health systems, and the development of agile surveillance systems is needed.

## Abstract 2

Jose Najera and colleagues identify lessons from the Global Malaria Eradication Programme (1955–1969) relevant to current elimination and eradication efforts.

**Summary Points**

- An examination of the evolution, implementation, and outcome of the Global Malaria Eradication Programme provides useful lessons for current elimination/eradication attempts
- Programmes should develop flexible strategies, integrated into the national health infrastructure rather than only implementing vertical malaria elimination campaigns, in order to ensure sustainability
- Professional cadres that can adapt the strategy to the local epidemiology and that can develop an effective surveillance system deeply rooted in the communities should be strengthened
- To solve problems and to review strategies, close links should be established with field and laboratory research
- Communities should be encouraged and supported to adopt malaria elimination as their own goal, reporting abnormal situations and creating a demand for effectiveness

## Introduction

The mechanisms of malaria transmission were first elucidated at the end of the 19th century. This research meant that malariologists could at last explain the observed effects of traditional control measures, such as drainage of marshes and mosquito nets, and develop better approaches to control malaria. Thanks to increasing public and political support, the early days of the 20th century witnessed the deployment of an increasing number of interventions against malaria. However, large-scale implementation of most of the proposed measures had severe operational and financial limitations, and some strategies were found to be suitable only in particular social, ecological, and epidemiological conditions.

The best approach to malaria control became the subject of intense debate during the first decades of the century. Experts were roughly divided into two major conceptual camps. Some (e.g., Ross, Gorgas, and Watson) favoured large-scale campaigns of vector control or mass drug administration to prevent and rapidly solve the problem. Others (the Malaria Commission of the League of Nations and the so-called Italian and Dutch schools) advocated locally designed programs of progressive, albeit slow, development of case management facilities and environmental sanitation to stimulate health and economic development, and diminish malaria morbidity and mortality. While the first group achieved spectacular successes, such as the interruption of malaria and yellow fever transmission during the construction of the Panama Canal and the elimination of the introduced highly efficient African vector *Anopheles gambiae* in Brazil, sustainability seemed to require the solid public health foundations envisaged by the second approach. Thus, in 1939 Boyd summarised the prevailing public health point of view as: “malaria control should not be a campaign, it should be a policy, a long-term program. It cannot be accomplished or maintained by spasmodic effort. It requires the adoption of a practicable program, the reasonable continuity of which will be sustained for a long term of years” [1].

It is hoped that the following review of the history of the Global Malaria Eradication Program (GMEP) (1955–1969) will encourage current and future antimalarial programmes that are pursuing new goals to develop flexible strategies on the basis of analyses of their own history and to strengthen their existing expertise rather than relying on new cadres to adopt an imported strategy, as did the GMEP.

## The Impact of DDT

The development of dichloro-diphenyl-trichloroethane (DDT) as the first residual insecticide in the early 1940s brought about a radical change in malaria control strategies. Killing indoor resting adult mosquitoes with insecticides sprayed on household walls had started in the 1930s using pyrethrum extracts, but had limited applicability because weekly applications were needed. DDT, which was first used against malaria by the US Army during World War II, required only semestrial or annual applications. This long residual effect meant that malaria control could be extended to large rural areas, although it needed a strong central organisation to handle the supply, transport, and distribution networks required for regular and correct application.

During the late 1940s and early 1950s, after numerous field trials, more and more national control programmes adopted DDT spraying. These programmes showed that transmission could be interrupted and that malaria did not necessarily return if spraying stopped [2],[3]. DDT appeared to be effective everywhere, making eradication of malaria a feasible objective. However, DDT's effectiveness against agricultural pests and household insects made prices soar, and its widespread application rapidly led to the first appearance of vector resistance to DDT in Greece in 1951 [4].

In this context, it was felt that progress at a global level would require more than the slow recruitment of political support country by country. Rather, it would be necessary to mobilise political commitment at the UN level and gain the financial support of UN agencies and of the United States, where a strong lobby was formed to obtain funds for global malaria eradication [5]. Further support for a global eradication approach was provided during the 1950s by Macdonald's mathematical model, which highlighted the great superiority of increasing adult vector mortality over mere reduction in density [6]–[8]. Malaria eradication was also advocated for with economic and political arguments that shifted from the impact of malaria on the local economies, to its influence on the price of imported goods and the risk that malaria could “predispose a community to infection with political germs that can delay and destroy freedom” as stated by Paul Russell, the Rockefeller malariologist who defended the WHO malaria eradication proposal at the 8th World Health Assembly (WHA) [9].

The GMEP was approved by the 8th WHA in Mexico in 1955 [10]. WHO was given the mandate to provide technical advice and coordinate resources, but not to act as “directing and coordinating authority” as proposed in the draft resolution submitted by 28 countries [11]. The 1955 WHA resolution also established a Malaria Eradication Special Account to channel public and private contributions [10], which opened the hope of general availability of funds.

Although approved by an overwhelming majority, the decision to launch the GMEP was not without controversy. Advocates of the eradication approach highlighted the emergence of mosquito resistance to DDT that, in their view, necessitated the launch of the GMEP before the world lost its most promising weapon. They also argued that eradication was, in the long term, financially more attractive than control. Conversely, critics of the campaign doubted the feasibility of eradication in vast areas that had poor communications and adverse environments and that lacked public health systems. They also emphasized the poor understanding of the implications of undertaking a malaria eradication campaign, both in terms of its cost and of the risk to the population posed by lost immunity if protection had to be interrupted [12].

**Figure 1:**
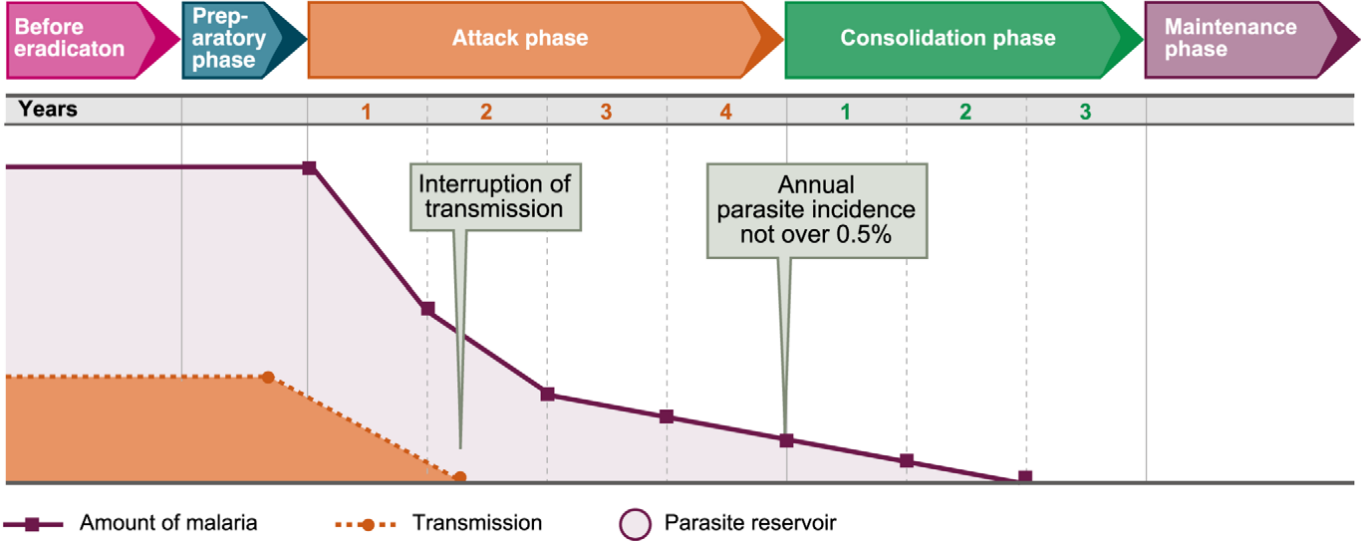
Phases of the Malaria Eradication Campaign as established by WHO in 1963. Image credit: Fusión Creativa.

In 1956, the WHO Expert Committee on Malaria was called to design the eradication campaign (Figure 1). The Committee felt that they were shaping a strong political force and that they had the opportunity of freeing malaria control from the frustrations of bureaucracy by prescribing autonomous organisations capable of achieving the precise execution of interventions. In contrast to control (measures of indefinite duration aimed at reducing the incidence of malaria), eradication was defined as “the ending of the transmission of malaria and the elimination of the reservoir of infective cases in a campaign limited in time and carried out to such a degree of perfection that when it comes to an end, there is no resumption of transmission” [13].

The Expert Committee seems not to have realised that in creating such executive machinery, they were transforming the practice of malariology. The administration of such gigantic enterprises was a totally absorbing job; up to then, malariologists had been field scientists guiding governments and local authorities by trying to solve a problem. Now malariologists were forced to become managers trying to accomplish a complex task.

Moreover, the global eradication campaign was based on the assumption that all the necessary knowledge for eradication was available, that further research was superfluous, and that eradication required a rigid discipline in which local deviations from a centrally defined plan must be prevented. Thus, malaria eradication acquired the characteristics of an ideology and control was demonised. This attitude is clearly illustrated by the statement of the UNICEF Regional Director to the Executive Board: “Between malaria control and eradication there is as great a difference as that between night and day. Control … is a primitive technique. Now we know exactly … the schedule of an eradication campaign which will last four or five years, followed by three years of consolidation” [14].

This overoptimistic environment prevented the recognition of general problems in the conception of the campaign, which was based on an exaggerated extrapolation of early local experiences that, although successful, represented a very limited variety of epidemiological situations. Actually, it was obvious from the start that nobody knew how to deal with the problems of tropical Africa; this was one of the main objections to the GMEP in the 1955 WHA.

A serious consequence of that exaggerated confidence was the belief that the wide experience and knowledge of the old malariologists was superfluous and even counterproductive, particularly if they persisted in modifying the eradication strategy locally. Therefore, eradication campaigns were entrusted to new, preferably young “malariologists,” trained in “Malaria Eradication Training Centres” established by WHO in several countries.

GMEP interventions consisted basically of indoor residual spraying with DDT or other approved insecticides. The Expert Committee developed standard guidelines for action on the basis of vertical, time-limited interventions clearly distinct from previous measures. Destruction of mosquito breeding marshes, prevention of mosquito bites, and other measures traditionally used in malaria control were abandoned, depicted not only as unnecessary but as antagonistic to the higher goal of eradication. Moreover, international funds became available only to countries adopting the goal and the means set by the WHO expert committee reports.

The fundamental principles of the campaign—total coverage and perfection in the execution of operations—served as a stimulus to those countries that already had, or could develop, the infrastructure to mobilize and use the new resources to eliminate malaria from their territories. Many other countries, following the Committee's directives, established new autonomous structures that favoured the delivery of services over the creation of a demand and the participation of local communities. These autonomous structures often became “self-perpetuating,” dissociated from the general health services and incapable of adaptation to changes in the epidemiological situation.

## Outcomes of the Campaign

It is not necessary to emphasize the positive contributions of the campaign to world health, which include: (1) achieving a considerable reduction in the geographical distribution of malaria although most of this reduction was in areas that already had well functioning control programmes; (2) being the first global health programme aimed at “total coverage”; (3) leading to the establishment, in some countries, of effective although partial contact with the communities, through networks of “voluntary collaborators” for diagnosis and treatment; (4) making a serious attempt to use local maps to guide its activities, even if that practice was later neglected; and (5) having an important influence on the subsequent planning of health programmes.

Nevertheless, as more and more countries joined the campaign and reported the achievement of total coverage with attack measures, often after strenuous efforts to reach remote areas, emerging problems were overlooked. Even the confirmation of chloroquine resistance in 1960, after treatment failures had been reported since the late 1950s from Venezuela and Thailand, was not given its full epidemiological importance because the campaign still hoped to interrupt transmission by spraying. In addition, it was assumed that the well-known periodic epidemic risk in certain areas would not return after local interruption of transmission. It was only in the mid-1960s that the existence of “problem areas” was recognised, after evidence of vector avoidance of contact with the insecticide in southern Mexico was confirmed.

As mentioned above, antimalarial interventions other than indoor residual spraying were abandoned. Even the use of antimalarial drugs as a complementary measure was considered redundant at the beginning. At the same time, there was a general disregard for social and cultural barriers, which often prevented the acceptance of the campaign activities in many of the “remote areas.” Moreover, even though most country programmes established health education units, these were rarely given the recognition or the means needed to provide a useful contribution [5].

## Malaria Resurgences after Interruption of Transmission

During the 1960s, not only did some areas fail to advance as expected, but other areas saw resurgences of malaria after relatively long periods of interruption of transmission. Some resurgences were surprisingly serious epidemics that required the reestablishment of spraying operations.

By 1962, it was already recognised that the consolidation phase required an infrastructure capable of supporting epidemiological surveillance. As a result, a new “pre-eradication programme” was established, mainly for Africa, with the aim of developing the required health infrastructure in parallel with the preparatory phase of the campaign. Unfortunately, there were no models of the minimum infrastructure required and the development of the “basic health services” continued to respond mainly to financial and political motivations.

Moreover, although by the mid-1950s, there was relatively wide experience in the use of DDT, nobody had a clear idea of how to organise a surveillance system capable of detecting the last cases of malaria. The sixth report of the Expert Committee [13] suggested the creation of surveillance systems involving direct—mainly house-to-house visits—and indirect means, such as engaging official or unofficial health services, of case detection. It also suggested that the search should be intensified as the number of cases decreased to manageable proportions.

However, the campaign managers considered terms like “manageable proportions” too vague and demanded clearer and more precise prescriptions. The Expert Committee obliged in its 8th and 10th reports by producing some indicators for when to stop total coverage with spraying (the end of the attack phase). These indicators were an annual parasite incidence of <0.5/1,000 and <0.1/1,000 (in the 8th and in the 10th reports, respectively), an annual blood examination rate of >10% of the population of the malarious areas, and a slide positivity rate of <5%. Although the committee insisted on the need to be guided by the experience and the capacity of the local services, campaign managers rapidly adopted these figures as thresholds for advancing through the phases of the campaign.

As problems became more widely recognised through the 1960s, there was some renewed interest in malaria research. WHO, for example, set up a programme for coordinating the development of new insecticides for public health and supported pilot projects to interrupt malaria transmission in Africa. Nevertheless, it was the spread of drug resistance in Southeast Asia and increased involvement of the US in the Vietnam war during the second half of the decade that led the US army to launch an intense malaria research programme aimed at the development of new antimalarials, but including studies on parasite biology, immune responses, in vitro culture, and the development of new animal models. McGregor described this development as: “throughout the world support for further research into malaria, even that concerned with insecticides and chemotherapeutics, contracted swiftly. Worse still, the apparent imminent demise of a once important disease removed the necessity for training scientists in malariology. It took 10 years and a war to halt this tragic trend” [15].

## After Global Eradication: A Return to Control

**Figure 2:**
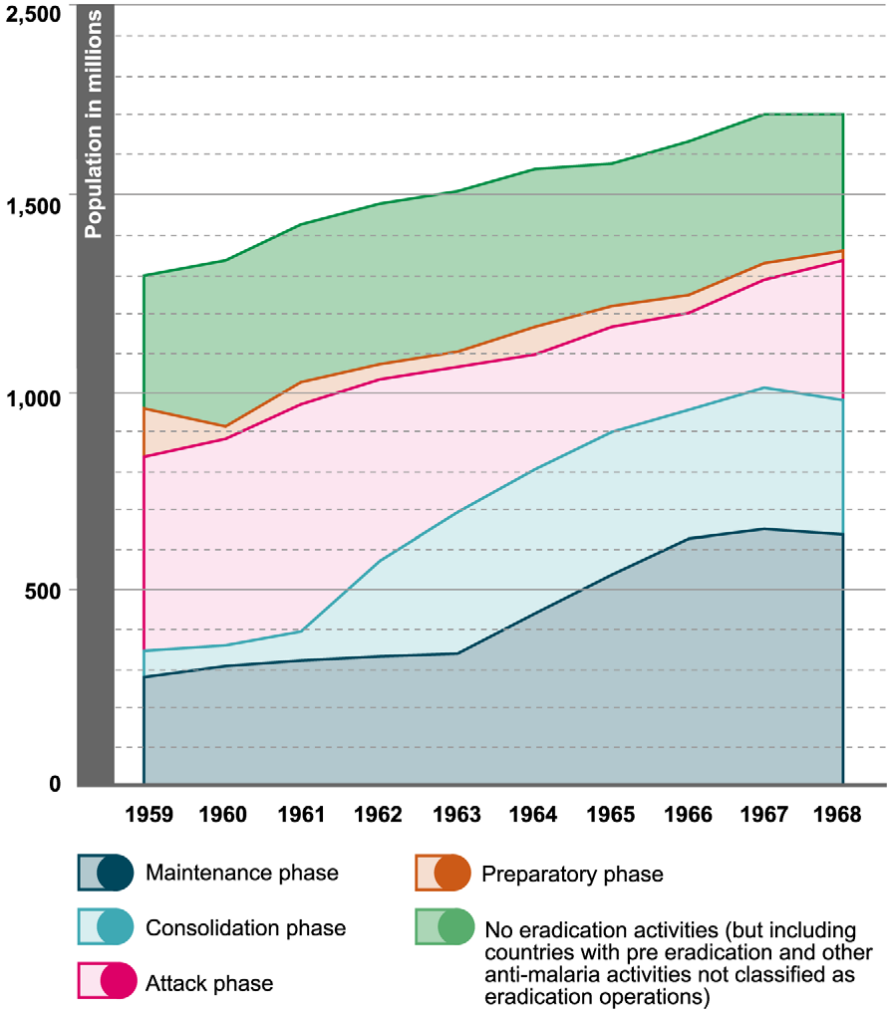
Progress of the campaign, presented to the 8th WHA [**16**]. Image credit: Fusión Creativa.

In 1967, as more areas reverted from consolidation to attack phase, the WHA requested a reexamination of the global strategy. The evaluation illustrated the slowing down of the global campaign [16], particularly after 1966 (Figure 2). GMEP also faced financial constraints during these years, as the US contributions to the WHO Malaria Special Account, which represented more than 85% of the total, were stopped in 1963, considerably reducing WHO's capacity to provide technical assistance [17].

An event that undoubtedly influenced the WHA was the 1968–1969 epidemic resurgence of malaria in Sri Lanka (then Ceylon), a country that had been considered a model for the training of malariologists. The surveillance system in this country had not reacted to 4 years of clear deterioration (1963–1967), nor had it taken into account 30 years of accumulated knowledge about the periodicity of epidemic risk in the country.

In 1969, 14 years after the launch of the GMEP, the 22nd World Health Assembly had to recognise that there were countries where eradication was not feasible in the short term, and that a strategy of control was an appropriate step towards future eradication in those areas. “In the regions where eradication does not yet seem feasible, control of malaria with the means available should be encouraged and may be regarded as a necessary and valid step towards the ultimate goal of eradication,” the Assembly stated, while reaffirming that eradication remained the ultimate objective [10].

## Malaria Control during the 1970s and 1980s

Faced with the recognition that malaria eradication could not be conceived as a short-term programme, UNICEF and other major collaborating agencies withdrew their support to malaria programmes in favour of general health programmes. The economic crisis of the early 1970s also contributed to the accelerated contraction of funding for malaria control. Moreover, oil shortages caused considerable increases in insecticide prices that further deteriorated the financial situation of the campaigns. This reduction of programme resources, aided by a strong La Niña in 1975–1976, resulted in severe epidemics in several countries, particularly in the Indian subcontinent and Turkey.

Another problem that became evident during the 1970s was the attrition of professional staff. The lack of professional incentives as a result of the routine work imposed during the GMEP had reduced the professional cadres. At the same time, the organisation of spraymen into unions made it increasingly difficult to reduce this unqualified labour force.

All these factors combined such that the campaigns became less and less capable of reorienting their strategy. This lack of flexibility, together with the drastic reduction of their operational capacity, led to the so-called “fire-fighting” strategy. Paradoxically, in the name of maintaining previous achievements, operations were continued in the best protected areas, resulting in resources being concentrated in the areas with lesser problems.

To make matters worse, in response to the economic crisis, many countries encouraged the exploitation of their natural resources. Some, like Brazil or Indonesia, actively supported the colonisation of their extensive primary forests by agriculture and mining, a process supported by the construction of penetrating roads. These policies resulted in massive outbreaks of malaria that, because of the relative weakness of official malaria control, encouraged an intensive trade of all kinds of antimalarial drugs, thus contributing to the spread of drug resistance [18].

All these problems supported the view that progress required the development of new tools and strategies and, in the mid 1970s, WHO launched the Special Programme for Research and Training in Tropical Diseases (TDR) in collaboration with the United Nations Development Programme and the World Bank, in an effort to reestablish the role of research in malaria control. Since its establishment, the TDR has achieved important successes in the development of new tools and in laboratory and field research.

Nevertheless, the “problem solving” approach of field malariologists in the first half of the 20th century has not been recovered in most programmes and the rift between control and research, once described in India as “a curious rivalry between the malaria programme and outside research bodies,” still persists. Most research projects have little operational bearing on the control programme and the latter lack “the capacity either to carry out research, to guide it, to generate issues for research based on analysis of incoming information, or to translate into operational use research carried out by other institutions” [18].

## Lessons Learnt from the GMEP by Antimalarial and Other Health Programmes

Throughout the past decades, countries have tried to adapt to changing situations within the constraints of their financial and organizational limitations. These experiences show how antimalarial and other programmes tried to implement lessons learned from the GMEP, even though there were sometimes great gaps between the formulation of a lesson and its application. These lessons included:

1. A public health service is needed to support malaria surveillance, even though there are still major disagreements among experts about when or how antimalarial programmes should be integrated with the health services. Relevant to this lesson, the WHO Registry of countries that have achieved local malaria eradication, elimination in present terminology, shows that a prerequisite for elimination may be the existence of a previous prolonged control programme that has contributed to the development of epidemiological services and a rural public health service (Table 1). Tourism-oriented, relatively rich islands maintain elimination through continuous expensive mosquito control programmes. It should also be recognised that countries included in the Registry were not highly malarious, although some of them had foci of high endemicity and areas subject to epidemic outbreaks.
2. Control has to be supported with research. This lesson has led to the considerable revival of malaria research since the 1970s, but the relations between control programmes and research institutions still need to be revived or strengthened.
3. As highlighted by the Primary Health Care movement, active participation of communities in the understanding of and actions for the solution of their health problems needs to be incorporated into antimalarial programmes. Although there have been important local initiatives in the past, WHO has only recently formulated a strategy for Community-based Malaria Elimination. Conversely, it is worth recalling that the setbacks and general lack of progress of the GMEP were among the main stimuli for the generation of the primary health care movement in the 1970s.
4. More specifically, the GMEP's “failure to achieve its objective” was taken into consideration in the design of the successful Intensified Smallpox Eradication Programme [19]. An important principle of this programme was that the administrative structure and pattern of operations of each national programme should be integrated into the health and socio-cultural setting of the country. Fenner and coauthors [20] noted that the programme's success depended on stating the strategic plan in terms of principles and illustrative methodologies rather than in terms of directives and on recognising that continuing field and laboratory research would be essential. Another important principle of the smallpox eradication programme was concentration on investigating all outbreaks or clustering of cases, before attempting to investigate each individual case. Although there are obviously great differences in the epidemiology and the response to control interventions between smallpox and malaria, these strategic considerations should now be taken into account in the malaria eradication programme where a lack of flexibility, an incapacity to adapt to changing situations, and a lack of coordination between control programmes and research institutions have all been identified as important obstacles to advancement in malaria control and elimination [17],[21],[22]. Noteworthy in this respect is China's experience. Although not included in the WHO Registry because only complete countries are included in this Registry, China has eliminated malaria from most of its territory by developing a control strategy on the basis of exhaustive attention to case detection and management by epidemiological services deeply rooted in their communal organisation. These services are firmly supported by political will at all levels of society and deploy well-organised control measures when they were needed for elimination of foci, all “in sensible semi-defiance of WHO dictates,” according to Kidson [23].

**Table 1:** Countries and regions certified malaria free up to 2010.

Countries and Regions with a Long History of Control	Islands with Tourism-Oriented Economy	Other
North Venezuela (1961), Hungary (1964), Spain (1964), Bulgaria (1965), Taiwan (1965), Cyprus (1967), Poland (1967), Romania (1967), Netherlands (1970), United States (1970), Italy (1970), Puerto Rico (1970), Cuba (1973), Portugal (1973), Yugoslavia (1973), Australia (1980), Turkmenistan (2010)	Grenada & Carriacou (1962), St. Lucia (1962) Trinidad & Tobago (1965), Dominica (1966), Jamaica (1966), U.S. Virgin Islands (1970), Mauritius (1973), Reunion (1979)	Singapore (1982), Brunei Darussalam (1987), United Arab Emirates (2007), Morocco (2010)

## Conclusions and Recommendations

Although not a comprehensive coverage of the problems of malaria control, the authors' experience and the broad historical considerations presented above, suggest the following conclusions, which may be useful in planning new elimination programmes:

- It may be fair to say that there is no country that is still endemic today where the malaria problem is so simple and uniform that it can be solved by applying a single strategy.
- The GMEP generated heated debate that contrasted vertical and horizontal approaches to malaria elimination. Historical analysis suggests that, while sustainable elimination of an endemic problem from a wide geographical area requires the build up of a epidemiological services well rooted in the communities, a well-organised, disciplined campaign is required for the rapid solution of local problems, such as outbreaks.
- It is essential to identify and study the physical, social, and cultural barriers that have proved to be stumbling blocks to malaria control in the past, and make all necessary efforts to avoid them in future by encouraging better community involvement and ownership.
- Programmes should be adequately integrated into the national health infrastructure. Such integration will allow them to benefit from available epidemiological services for communication and analysis. Programmes should also benefit from the establishment of solid links with research and training institutions, including organisations studying ecology, anthropology, sociology, economic activities (e.g., agriculture, forestry, mining, fishing, etc.), production systems, labour relations, and population movements of endemic populations.
- Worryingly, the notion that problems can be solved before they are fully understood still seems widespread. This attitude is evidenced by the emphasis placed on scaling up control interventions rather than on developing an epidemiological infrastructure. While such scaling-up will most likely continue to reduce transmission in many areas, the timely identification and elimination of residual foci may not be possible unless programmes reestablish strong professional cadres capable of guiding flexible and adaptable action. That is, those involved in elimination efforts need to not only apply accepted control measures, but also to evaluate results and participate in problem solving.
- Surveillance should not only aim to detect the last case, it should be an essential instrument from the start, involved in the identification and study of problem areas, beyond the limits of administrative localities. As the elimination programme advances, epidemiological investigations should concentrate successively in the study of outbreaks or clustering of cases and finally of individual case investigations.

Finally, it is necessary to break the “quasi-cyclical” alternation between overoptimistic expectations and a “fire-fighting strategy.” If malaria eradication is ever to succeed, the fate stated in 1927 by the Second Report of the Malaria Commission of the League of Nations—“The history of special antimalarial campaigns is chiefly a record of exaggerated expectations followed sooner or later by disappointment and abandonment of the work”—must be avoided.

## Abbreviations

GMEP: Global Malaria Eradication Program
DDT: dichloro-diphenyl-trichloroethane
WHA: World Health Assembly

## References

[1] Boyd MF (1939). Malaria: Retrospect and prospect.. Am J Trop Med Hyg.

[2] Livadas GA (1952). Is it necessary to continue indefinitely DDT residual spraying programmes? Relevant observations made in Greece. WHO document WHO/MAL/79..

[3] Pampana E (1969). A textbook of malaria eradication. 2nd edition..

[4] Livadas GA, Georgopoulos G (1953). Development of resistance to DDT by *Anopheles sacharovi* in Greece.. Bull World Health Organ.

[5] Cueto M (2007). Cold war, deadly fevers. Malaria eradication in Mexico, 1955–1975..

[6] Macdonald G (1957). The epidemiology and control of malaria..

[7] MacDonald G (1956). Epidemiological basis of malaria control.. Bull World Health Organ.

[8] The malERA Consultative Group on Modeling (2011). A research agenda for malaria eradication: Modeling.. PLoS Med.

[9] Russell PF (1955). Man's mastery of malaria..

[10] WHO (1973). Malaria. Handbook of resolutions and decisions of the World Health Assembly and the Executive Board. Volume I, 1948–1972, 1st to 25th WHA and 1st to 50th EB..

[11] Gramiccia G, Beales PF, Wernsdorfer WH, McGregor I (1988). The recent history of malaria control and eradication.. Malaria. Principles and practice of malariology.

[12] WHO (1955). Eighth World Health Assembly (Mexico, D.F., 10–27 May 1955). Official records of the World Health Organization, N° 63..

[13] WHO (1957). Expert Committee on malaria, sixth report, WHO Technical Report Series, number 123..

[14] UNICEF (1955). Statement read by the Regional Director before the Executive Board at its September Meeting. The Americas Regional Office Programme Progress Report, number 29 (March-August, 1955)..

[15] McGregor IA (1982). Malaria: Introduction.. Br Med Bull.

[16] WHO (1969). Re-examination of the global strategy of malaria eradication. Twenty-second World Health Assembly, Part I. WHO official records number 176, annex 13..

[17] Nájera JA (2001). Malaria control: achievements, problems and strategies.. Parassitologia.

[18] Evaluation Committee (1985). In-depth evaluation report of Modified Plan of Operation under National Malaria Eradication Programme of India..

[19] Henderson DA (1998). Eradication: Lessons from the past.. Bulletin of the World Hlth Organization.

[20] Fenner F, Henderson DA, Arita I, Ježek Z, Ladnyi ID (1988). Smallpox and its eradication..

[21] Greenwood BM (2008). Control to elimination: Implications for malaria research.. Trends Parasitol.

[22] Breman JG (2004). Conquering the intolerable burden of malaria: What's new, what's needed: a summary.. Am J Trop Med Hyg.

[23] Kidson C, Indaratna K (1998). Ecology, economics and political will: The vicissitudes of malaria strategies in Asia.. Parassitologia.

